# Computed tomography morphometric analysis of the greater palatine canal: a study of 1,500 head CT scans and a systematic review of literature

**DOI:** 10.1007/s12565-014-0263-9

**Published:** 2014-12-03

**Authors:** Iwona M. Tomaszewska, Elizabeth K. Kmiotek, Iwona Z. Pena, Michał Średniawa, Katarzyna Czyżowska, Robert Chrzan, Michał Nowakowski, Jerzy A. Walocha

**Affiliations:** 1 Department of Medical Education, Jagiellonian University Medical College, 16 Lazarza Street, 31-530 Krakow, Poland; 2 Department of Anatomy, Jagiellonian University Medical College, Krakow, Poland; 3 Department of Radiology, Jagiellonian University Medical College, Krakow, Poland

**Keywords:** Canal, Greater, Palatine, Morphometric analysis, Pterygopalatine, Systematic review

## Abstract

We have performed a detailed morphometric analysis of the length and anatomic routes of the greater palatine canal (GPC) and a systematic review of the literature on the anatomy of the GPC with the aim of informing dentists, maxillofacial surgeons, otorhinolaryngologists and other specialists performing procedures in the area of the GPC. In total, we analysed 1,500 archived adult head computed tomography scans to determine the length of the GPC and of the routes on both sides, as well as the dimensions and opening directions of the greater palatine foramen. The systematic review of the literature was performed according to PRISMA guidelines. The study group comprised 783 females (52.2 %) and 717 males with a mean (± standard deviation) age of 42.1 ± 16.9 years; there was significant difference in age between sexes (*p* = 0.33). The average length of the GPC was 31.1 ± 2.9 (range 15–44) mm. The GPC travelled three different paths in the sagittal plane and four different paths in the coronal plane. Most often it descended from the pterygopalatine fossa inferiorly before changing to an anterior-inferior direction (68.4 %; sagittal plane) and inferior-laterally before changing to an inferior-medial direction (40.7 %; (coronal plane). In total, the GPF had four different opening directions: inferior-anterior-medial (82.1 %), inferior-anterior-lateral (4.0 %), anterior (7.6 %), and vertical (5.3 %). Twenty-five studies were included in the systematic review. In conclusion, the information presented here provides clinicians with the anatomical knowledge necessary to minimize the risk of complications when performing procedures involving infiltration of the GPC.

## Introduction

The greater palatine canal (GPC) communicates with the oral cavity through the greater palatine foramen (GPF), which is most commonly located opposite the third molar (Tomaszewska et al. 2014a). The GPC continues in a posterior-superior direction, terminating at the pterygopalatine fossa (PPF) which is an inverse pyramid-shaped space communicating with the middle cranial fossa via the foramen rotundum, the nasal cavity via the sphenopalatine foramen, the orbit via the inferior orbital fissure and the oral cavity via the GPF (Erdogan et al. 2003). The walls of the PPF are formed anteriorly by the infratemporal surface of the maxilla, posteriorly by the pterygoid process of the sphenoid and medially by the perpendicular plate of the palatine bone. The GPC itself passes through the palatine bone (Howard-Swirzinski et al. 2010). The GPC houses the descending palatine artery and the greater and lesser palatine nerves as well as their posterior inferior lateral nasal branches, while the PPF contains the maxillary artery and its branches, the accompanying vein, the maxillary nerve and its branches and the pterygopalatine ganglion (Hwang et al. 2011).

The anatomy of these structures is of great importance to dentists, maxillofacial surgeons, otorhinolaryngologists and other specialists performing medical procedures in the area of the GPC. Using the GPC approach to the PPF clinicians are able to achieve a maxillary division nerve block (for dental or maxillofacial procedures), haemostasis (for endoscopic sinus surgery, septorhinoplasty or to control posterior epistaxis) and/or relief of sphenopalatine neuralgia (Das et al. 2006; Douglas and Wormald 2006; McKinney et al. 2010). The blocking of sensation of the maxillary nerve in the PPF achieves anaesthesia of the maxillary teeth, the maxillary palatal and gingival tissue, as well as of the skin of the midface, nasal cavity and sinus (Sharma and Garud 2013). However, due to the close relationship of the anatomical structures inside the GPC and the PPF, as well as the direct communication of the PPF with the inferior orbital fissure, infiltration of the PPF through the GPC may result in complications. These include intravascular or intracranial injection, infraorbital nerve injury, transient ophthalmoplegia, diplopia, ptosis, neural tissue damage, intracranial infection and/or even blindness from vasoconstriction of the ophthalmic artery (Das et al. 2006; Douglas and Wormald 2006).

To successfully produce a maxillary nerve block and to minimize the risk of complications, clinicians require a thorough knowledge of GPC anatomy. The anatomy of the GPC has been investigated from the middle of the 20th century onwards (Viegas and Hemphill 1961; Das et al. 2006), but it was only after the introduction of computed tomography (CT) that detailed analyses became possible (Das et al. 2006; Douglas and Wormald, 2006; Howard-Swirzinski et al. 2010; McKinney et al. 2010; Hwang et al. 2011; Sheikhi et al. 2013). However, even though several CT studies evaluating GPC anatomy have been carried out to date, a unified range of values of GPC is still lacking in the literature. Additionally, the authors of all of the abovementioned studies base their conclusion on study groups of small or moderate size, which prevents the drawing of definitive conclusions on the optimal injection depth when using the GPC.

We therefore undertook the present study to obtain morphometric details on both the length and anatomic routes of the GPC in a large sample of head CT scans. We also performed a systematic review of the literature on GPC anatomy to improve the anatomical knowledge of clinicians in a unified manner. This latter aim was achieved by extracting relevant measurements from each study in the review and comparing these in the form of a table.

## Materials and methods

### Study material

Preliminary screening was conducted on 6,471 archived adult Caucasian head CT scans (Department of Radiology, Jagiellonian University Medical College and Department of Radiology, J. Dietl’s Specialistic Hospital, Krakow, Poland), of which 1,500 (23.2 %) CT scans met the study inclusion criteria and formed the basis for conducting measurements.

 The CT images were acquired using a Somatom Sensation 16 scanner (Siemens Healthcare, Erlangen, Germany) and an Aquilion 64 scanner (Toshiba Medical Systems, Tokyo, Japan). The following study parameters were applied:exposure 120 kV, 74 mA, 60 mAs; rotation time 0.5 s; slice thickness 0.5 mm. Patient’s sex and age data were acquired from patient files.

 Study inclusion criteria were full eruption of third molars on both sides of the maxilla, presence of all maxillary teeth, patient age of >21 years and absence of any pathological (including developmental and traumatic) changes in the region of the maxilla.

### Measurements

The measurements (CT scans) were performed using the eFilm Workstation 3.4 (Merge Healthcare, Chicago, IL). Maximum intensity projections, multi-planar reconstructions and volume rendering reconstructions were examined in three planes—coronal, sagittal and transverse. All measurements were recorded to the nearest 0.01 mm and, after statistical analysis, were rounded off to the nearest 0.1 mm for data presentation. All bilateral measurements were performed symmetrically. Each measurement was taken twice by the same observer; in the case of any discrepancies, the mean of the two values was recorded. Following measurements of all scans, randomly chosen samples (20 % of original number) were re-measured by an observer who did not partake in the first assessment of the scans. Inter-class correlations (ICC) were calculated, and the level of agreement between the assessments was very high (ICC = 0.93–0.96).

The centre of the GPF was established while measuring its anterior-posterior (AP) and lateral-medial (LM) dimensions, as described by Tomaszewska et al. (2014a). The centre of the GPF was set at the point of the intersection of two straight lines representing the longest AP and LM GPF dimensions. If necessary, this intersection was corrected visually using the GPF form factor. The form factor was obtained by dividing the AP GPF dimension by the LM dimension. If the GPF was circular in shape, the obtained value was equal to 1; values of >1 indicated that the GPF was elongated in the AP dimension, and values of <1 indicated that the foramen was elongated in the LM dimension (Jaffar and Hamadah 2003).

**Fig. 1:**
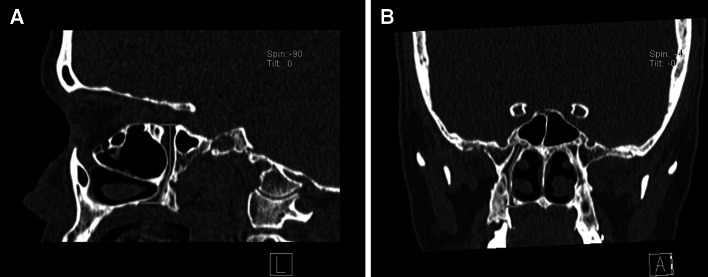
Greater palatine canal (GPC) length measurements in both the sagittal (**a**) and coronal (**b**) planes. The *red line* depicts the route by which the GPC was measured (color figure online)

The following assessments were performed:

1. GPC length on both the right (R) and left (L) sides. The length of the GPC was measured according to the methodology of Howard-Swirzinski et al. (2010) in both the sagittal and coronal planes (Fig. 1). The measurements from both planes were then averaged to obtain the final GPC length. The superior aspect of the GPC was set at the centre of the pterygoid canal (the centre point of the PPF). The inferior aspect of the GPC was marked at the inferior surface of the hard palate. In the sagittal plane, the GPC was measured from the centre point of the PPF (superior aspect) to the posterior wall of the GPF (inferior aspect). In the coronal plane the GPC was measured from the centre point of the PPF (superior aspect) to the inferior surface of the horizontal hard palate for standardization due to variance in the shape of the foramen (inferior aspect). The GPC was measured in millimeters using the straightest linear path passing through the centre of the canal.

2. GPC route in sagittal and coronal planes (qualitative assessment combined with quantitative analysis of angles relating to GPC directional changes). Angles given in the Results section represent the deviation from a theoretical line vertical to the long axis of the body.
3. Evaluation of the opening direction of the GPF (qualitative assessment combining GPC direction near its GPF end, bone level between the alveolar ridge and the palatine bones in the coronal plane and GPF opening direction in the sagittal plane).
4. Evaluation widest AP and LM dimensions of the GPF.

### Literature search

**Fig. 2:**
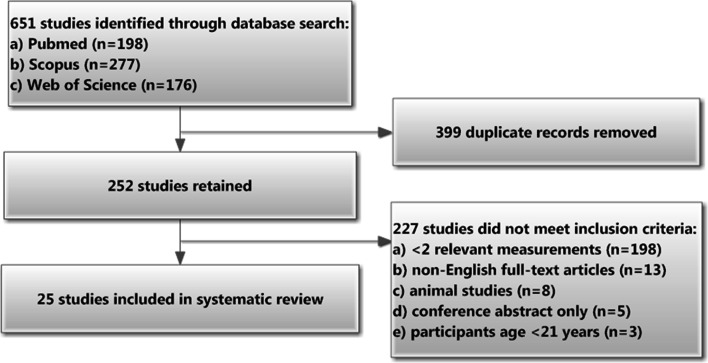
Flowchart depicting literature search and study selection

In the literature search we strictly adhered to the Preferred Reporting Items for Systematic Reviews and Meta-Analysis (PRISMA) guidelines (http://www.prisma-statement.org/2.1.2%20-%20PRISMA%202009%20Checklist.pdf). The search process is shown as a flowchart in Fig. 2. Two independent reviewers searched the PubMed, Scopus and Web of Science databases for appropriate studies published up to 1 July 2014 (no lower date limit) using the search keywords “greater”, “palatine”, “canal”, “pterygopalatine” and “foramen” in different combinations, as per Boolean logic rules. Review of full-text articles was limited to those published in English. References of identified articles were searched manually. Study inclusion criteria were (1) studies conducted on human skulls/head CT scans; (2) participants aged ≥21 years; (3) full-text original articles only (excluding conference abstracts and review papers); (4) ≥2 relevant measurements. Inclusion or exclusion of studies was performed hierarchically based on the title of the report first, followed by the abstract and finally by the full text.

Data extraction and quality assessment was performed independently by two reviewers. The following data were extracted from the relevant studies: citation details, sample size, sample characteristics and relevant measurements performed.

### Comparison of relevant measurements

The measurements extracted from each study included in the literature search and those obtained in the present study were compared, including GPF opening direction, GPF dimensions and mean GFP length.

### Statistical analysis

Statistical analysis was conducted using STATISTICA 10 PL (StatSoft Inc., Tulsa, OK). Elements of descriptive statistics were used [mean, standard deviation (SD), percentage distribution]. Side-related differences were evaluated using the Student’s *t* test. The ICC was used to evaluate the level of agreement between measurement and re-measurement of the same sample. A *p* value of <0.05 was considered statistically significant.

### Ethics

This study has been approved by the Jagiellonian University Medical College Bioethics Committee (registry no KBET/161/B/2013) and was performed in accordance with the ethical standards laid down in the 1964 Declaration of Helsinki and its subsequent amendments.

## Results

The study group comprised 1,500 patients (783 female; 52.2 %) for whom head CT scans were available for analysis, yielding a total of 3000 GPC for further evaluation. The mean age of the group was 42.1 ± 16.9 years, and there was no age difference between sexes (*p* = 0.33). The average length of the GPC was 31.1 ± 2.9 (range 15–44) mm. The results of the main measurements are summarized in Table 1.

**Table 1:** Results of main measurements^a^

Measurement	Right side	Left side	*p* value right vs. left	Total	*p* value male vs. female
GPC length (male) (*n* = 717)	32.6 (2.8)	32.4 (2.8)	0.18	32.5 (2.8)	<0.0001
GPC length (female) (*n* = 783)	29.6 (2.5)	29.9 (2.7)	0.02	29.9 (2.6)	
GPF AP dimension (male)	5.1 (0.5)	5.1 (0.4)	1.00	5.1 (0.4)	<0.0001
GPF AP dimension (female)	5.0 (0.4)	5.0 (0.4)	1.00	5.0 (0.4)	
GPF LM dimension (male)	3.0 (0.7)	2.9 (0.5)	0.002	2.9 (0.6)	0.007
GPF LM dimension (female)	2.8 (0.8)	2.8 (0.8)	1.00	2.8 (0.8)	

*SD* standard deviation, *GPC* greater palatine canal, *GPF* greater palatine foramen, *AP* anterior-posterior, *LM* lateral-medial

^a^Results of main measurements are presented as the mean, with the standard deviation (SD) in parenthesis

In total, the GPF had four different opening directions, namely, inferior-anterior-medial (82.1 %), inferior-anterior-lateral (4.0 %), anterior (7.6 %) and vertical (5.3 %). Detail on the opening direction analysis of the GPF is presented in Table 2.

**Table 2:** Incidence of the opening directions of the greater palatine foramen

Opening direction	GPC (right side) (%)	GPC (left side) (%)	Bilaterally symmetrical (%)	Overall incidence (%)
Inferior-anterior-medial	81.3	77.1	79.2	82.1
Inferior-anterior-lateral	82.7	83.3	83.0	4.0
Anterior	68.1	61.3	64.7	7.6
Vertical	74.3	77.5	75.9	5.3

Overall number of GPC for analysis = 3,000 (right side = 1,500; left side = 1,500)

The GPC travelled three different paths in the sagittal plane and four different paths in the coronal plane.

### Sagittal plane

**Fig. 3:**
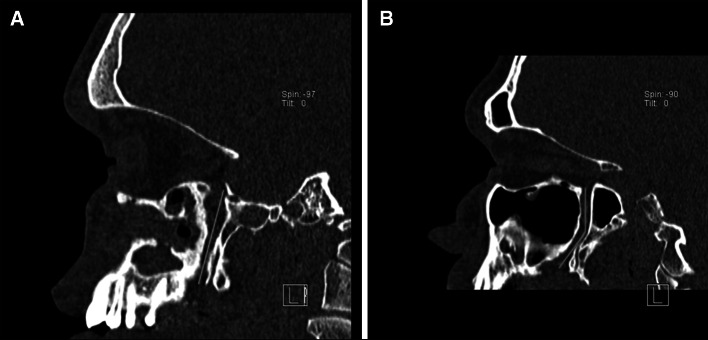
Types of pathways of the GPC observed in the sagittal plane. **a** GPC travels in an anterior-inferior direction from the pterygopalatine fossa (PPF), **b** GPC first travels in an inferior direction, then in an anterior-inferior direction through the remainder of the canal. The *red line* depicts the pathway of the GPC (color figure online)

- The GPC travels in an anterior-inferior direction from the PPF (30.4 %) (Fig. 3a).
- The GPC first travels in an inferior direction and then in an anterior-inferior direction through the remainder of the canal (68.4 %) (Fig. 3b).
- Other (1.2 %).

### Coronal plane

**Fig. 4:**
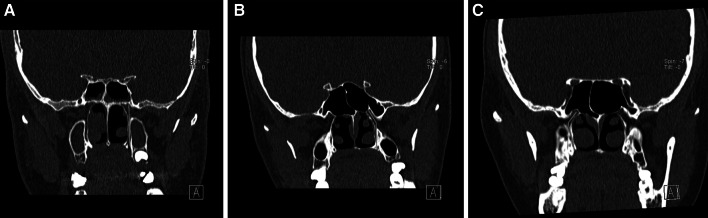
Types of pathways of the GPC observed in the coronal plane. **a** GPC travels directly in a inferior direction from the PPF, **b** GPC travels in an inferior-lateral direction from the PPF, then directly inferior, **c** GPC travels in an inferior-lateral direction from the PPF, then changes to an inferior-medial direction for the remainder of the canal. The *red line* depicts the pathway of the GPC (color figure online)

- The GPC travels in a directly inferior direction from the PPF (17.6 %) (Fig. 4a).
- The GPC travels in an inferior-lateral direction from the PPF and then directly inferior (39.9 %) (Fig. 4b).
- The GPC travels in an inferior-lateral direction from the PPF and then changes to an inferior-medial direction for the remainder of the canal (40.7 %) (Fig. 4c).
- Other (1.8 %).

The incidences of the different GPC paths are summarized in Table 3, and the average angles and directional distances are summarized in Table 4. There was a statistically significant difference in GPC length between patients with GPC of an directly inferior pathway type (coronal plane) and those with GPC of an “alternating” (any other than directly inferior) pathway type (30.8 ± 3.2 vs. 31.2 ± 2.8, respectively; *p* = 0.04).

**Table 3:** Incidence of greater palatine canal pathways in both the sagittal and coronal planes

Pathway type	GPC (right side) (%)	GPC (left side) (%)	Bilaterally symmetrical (%)	Overall incidence (%)
Sagittal plane				
Anterior-inferior (Fig. 3a)	30.1	30.7	73.0	30.4
Inferior > anterior-inferior (Fig. 3b)	69.5	67.2	81.8	68.4
Other	0.9	1.6	0	1.2
Coronal plane				
Inferior (Fig. 4a)	18.7	16.4	7.3	17.6
Inferior-lateral > inferior (Fig. 4b)	38.9	40.9	17.3	39.9
Inferior-lateral > inferior-medial (Fig. 4c)	40.1	41.3	29.0	40.7
Other	2.3	1.4	0.1	1.8

Overall number of GPC for analysis = 3,000 (right side = 1,500; left side = 1,500)

**Table 4:** Average angles and directional distances of observed greater palatine canal pathway pathways

Pathway type	Directional distance	GPC (right side)	GPC (left side)
Sagittal plane			
Anterior-inferior (Fig. 3a)	Anterior-inferior angle (°)	28.4 (3.5)	28.2 (3.5)
Inferior- >anterior-inferior (Fig. 3b)	Directly inferior distance (mm)	9.4 (3.2)	8.7 (4.1)
	Anterior-inferior angle (°)	31.1 (4.3)	31.0 (4.5)
Coronal plane			
Inferior-lateral- >inferior (Fig. 4b)	Inferior-lateral angle (°)	28.5 (5.0)	28.2 (5.0)
	Inferior-lateral distance (mm)	6.9 (1.7)	7.1 (2.0)
Inferior-lateral- >inferior-medial (Fig. 4c)	Inferior-lateral angle (°)	26.3 (1.8)	27.2 (2.5)
	Inferior-lateral distance (mm)	9.9 (4.0)	10.6 (3.6)
	Inferior-medial angle (°)	13.1 (4.7)	13.1 (4.2)

The straight inferior pathway viewed in the coronal plane is not included because those canals followed a direct vertical path

Data are presented as the average, with the SD in parenthesis

The flowchart depicted in Fig. 2 presents the results of the literature search. Of the studies identified during the initial literature search (*n* = 651) only 25 studies were included in the final systematic review (24 studies identified from the literature search and the present study). Table 5 compares data on GPF and GPC measurements from these 25 studies.

**Table 5:** Comparison of selected parameters from the 25 studies^a^ included in the systematic review

Study	Population (number of samples)	Type of investigation and sample characteristics	GPF opening direction (%)				GPF dimensions (mm)		Mean GPC length (mm)	
			I-A-M	I-A-L	Anterior	Vertical	AP	LM		
Tomaszewska et al. (2014) (this study)	Polish (*n* = 1500)	Sinus CT scans; mean age 42.1 ± 16.9; total: 717 M, 783 F	82.1	4.0	7.6	5.3	5.0 (0.4)	2.9 (0.7)	31.1 (2.9)^b^; range 15–44	
Nimigean et al. (2013)	Romanian (*n* = 100)	Dry human skulls; age range 25–40; sexed	82.0	–	13.0	5.0	4.9 (0.9)	3.0 (0.9)	–	
Piagkou et al. (2012)	Greek (*n* = 71)	Dry human skulls; adult; unsexed	–	–	–	–	5.3 (0.9)	2.7 (0.5)	–	
Howard-Swirzinski et al. ( 2010)	American (*n* = 500)	CBCT scans; age range 18–73; total: 235 M, 265 F	–	–	–	–	–	–	29.0 (3.0)^b^; range 22–40	
McKinney et al. (2010)	American (*n* = 10)	Maxillofacial CT scans; age range 18–64; unsexed	–	–	–	–	–	–	40.4 (1.9)^b^	
Das et al. (2006)	American (*n* = 100)	Sinus HRCT scans; adult; total: 50 M, 50 F	–	–	–	–	–	–	SPF-GPF distance: M: 28 ± 2 range 27–29; F: 27 ± 2 range 25–29	
Osunwoke et al. (2011)	Nigerian (*n* = 150)	Dry human skulls; adult; sexed (100 % M)	–	–	–	–	–	15.0 (2.1)	–	
Ajmani (1994)	Nigerian (*n* = 65)	Dry human skulls; adult, unsexed	58.7	38.7	–	–	–	–	–	
Hassanali and Mwaniki (1984)	Kenyan (*n* = 125)	Dry human skulls; adult; total: 60 M, 22 F, 43 unsexed	74.0	–	–	26	–	–	–	
Langenegger et al. (1983)	South African (*n* = 100)	Dry human skulls; mean age 42.7; total: 50 M, 50 F	–	–	–	–	–	2.5 (0.5)	–	
Hwang et al. (2011)	Korean (*n* = 50)	Head HRCT scans; mean age 51.0; total: 22 M, 28 F	–	–	–	–	4.5 (0.7)	2.2 (0.4)	34.8 (2.7)^b^; range 22.6–48.5	
Klosek and Rungruang (2009)	Thai (*n* = 41)	Human cadavers; mean age 71.2; total: 24 M, 17 F	–	–	–	–	F: 5.1 (1.0) M: 4.9 (8.3)	F: 2.8 (1.0) M: 2.6 (8.3)	–	
							3.25^a^ (0.5)			
Methathrathip et al. (2005)	Thai (*n* = 160)	Dry human skulls (*n* = 105) – mean age 48.1; total: 68 M, 37 F; human cadavers (*n* = 55)	–	–	–	97.6	4.9 (0.9)	2.7 (0.5)	M: 30.0 (4.3) range 16.3–40.9; F: 27 ± 2 range 25–29	
									29.7 (4.2)^b^; range 16.3–40.9	
Wang et al. (1988)	Chinese (Taiwan) (*n* = 100)	Dry human skulls; adult; sexed	–	–	90.0	10.0			–	
Ikuta et al. (2013)	Brazilian (*n* = 50)	CBCT scans; mean age 35.8; total: 27 M, 23 F	**–**	**–**	**–**	**–**	3.1^a^ (0.5)		–	
Chrcanovic and Custódio (2010)	Brazilian (*n* = 80)	Dry human skulls; age unknown; unsexed	18.8	0.0	69.4	11.9	–	–	–	
Dave et al. (2013)	Indian (W) (*n* = 100)	Dry human skulls; adult; total: 60 M, 39 F, 1 U	–	–	4.0	96.0	–	–	–	
Sharma and Garud (2013)	Indian (W) (*n* = 100)	Dry human skulls; adult; unsexed	49.5	3.5	2.0	45.0	4.7 (1.1)	3.25^b^ (0.5)	–	
Kumar et al. (2011)	Indian (N) (*n* = 100)	Dry human skulls; adult; unsexed	19.0	73.0	1.0	7.0	–	–	–	
Saralaya and Nayak (2007)	Indian (SW) (*n* = 132)	Dry human skulls; adult; unsexed	46.2	12.5	41.3	–	–	–	–	
Ajmani (1994)	Indian (N) (*n* = 34)	Dry human skulls; adult, unsexed	91.4	–	–	–	–	–	–	
Westmoreland and Blanton (1982)	Indian (E) (*n* = 300)	Dry human skulls; adult; unsexed	–	–	18.0	82.0	–	–	–	
Sheikhi et al. (2013)	Iranian (*n* = 138)	CBCT scans; age range 18–76; total: 73 M, 65 F	–	–	–	–	–	–	Right-side M: 32.7 (2.5) F: 30.5 (1.7); Left-side M: 33.2 (2.3) F: 30.6 (1.7)	
									31.8 (1.4)^b^	
Douglas and Wormald (2006)	Australian (*n* = 21)	Head CT scans; formalin fixed cadavers; mean age 81 ± 8.9; total: 13 M, 8 F	–	–	–	–	–	–	40.1^b^; range 38.6–41.6	
Jaffar and Hamadah (2003)	Caucasian (Iraqi) (*n* = 50)	Dry human skulls; adult; unsexed	60.0	–	36.0	4.0	4.6 (1.0)	2.8 (0.6)	–	
Malamed and Trieger (1983)	Mixed (*n* = 204)	Dry human skulls; adult; unsexed	–	–	38.7	61.3	–	–	–	

Data are presented as the mean, with the SD in parenthesis where appropriate

*CT* Computed tomography,* CBCT*, cone beam CT, *HRCT* high-resolution computed tomography, *M* male, *F* female, *I*-*A*-*M* inferiorly antero-medially*, I*-*A*-*L* inferiorly antero-laterally, *SPF* sphenopalatine foramen, *N* north, *S* south, *E* east, *W* west

This table presents the data from 25 studies (24 identified in the literature search and the present study) containing relevant measurements. The study population from the work of Ajmani (1994) has been divided in two, as the study analyses two different populations (African and Indian) (hence the 26 studies in the table)

^a^GPF diameter

^b^Total

## Discussion

The GPC approach can be used to achieve a maxillary nerve block and to minimize bleeding during endoscopic sinus surgery or septorhinoplasty. However, avoidance of the potential complications that can arise during this procedure require a thorough knowledge of the anatomy of the surrounding structures. Our study has shown that in a eastern European population the GPC has an average length of 31.1 ± 2.9 mm, with a range of 15 to 44 mm. The GPC most often descends from the PPF inferiorly, then changing to an anterior-inferior direction (sagittal plane), and inferior-laterally, then changing to an inferior-medial direction (coronal plane). The GPF most often opens in an inferior-anterior-medial direction.

Recent technological developments will surely increase the importance of an adequate knowledge of GPC anatomy. For example, Piagkou et al. (2012) reported that the pterygopalatine ganglion can be stimulated through the GPF and GPC to reduce the effect of stroke in stroke patients. Other researchers have highlighted the role of the pterygopalatine ganglion in cerebrovascular autonomic physiology, in the pathophysiology of cluster and migraine headaches and in conditions of cerebral vasospasm (Oluigbo et al. 2011).

The suggested recommended length of insertion of the anaesthetic needle into the GPC ranges between 25 mm (haemostasis) to 39 mm (maxillary nerve anaesthesia) (Wong and Sved 1991; Das et al. 2006; Douglas and Wormald 2006). Table 5, which presents the results of our systematic review of literature, allows for easy comparison between studies in terms of GPC length and GPF opening direction. The results of most of the studies included in the review fall into the range of data reported in the present study. However, there are a number of outliers, such as the studies by McKinney et al. (2010) (mean GPC length 40.4 mm), Douglas and Wormald (2006) (mean GPC length 40.1 mm) and Hwang et al. (2011) (mean GPC length 34.8 mm). In the case of the first two studies, the most probable reason for the GPC length discrepancy is the small size of the study cohort, which did not exceed ten and 21 subjects, respectively. Regarding the study by Hwang et al. (2011), the deviation from the overall GPC length trend is smaller than that of the other two studies and may be attributed to the ethnicity of the sample studied.

The anterior-posterior and lateral-medial dimensions of the GPF proved to be similar among the studies included in the review, even between those with different subject ethnicity. However, it proved to be difficult to compare the opening direction of the GPF among the studies—not only due to the different estimation methods used by the authors but also because some measurements were based on dry skulls as well as head CT scans. Wang et al. (1988) suggested that these differences might originate from racial variations between the examined subjects, but we suggest that the discrepancies are purely the result of employing different estimation techniques. We specifically use the term “estimation” as the only sure way to determine the opening direction of the GPF is by basing the measurement on coronal and sagittal head CT scans. Manual assessment of dry skulls only approximates GPF opening direction. Though vertical openings were a rare finding in our group, their presence may explain the occasional clinical difficulty encountered when attempting to insert the needle point into the GPC. Additionally, according to Slavkin et al. (1966), the frequency of anatomical obstruction of the needle in the GPC increases with age.

Several methods of infiltrating the PPF have been described, but the most widely accepted GPF injection technique is that proposed by Stankiewicz (1988). Authors describing PPF infiltration for anaesthetic purposes recommend bending the needle at the hub to a 30° angle and then advancing it by about 38 mm (Mercuri 1979). This allows for deposition of the anaesthetic close to the infraorbital nerve, thus producing reliable anaesthesia (Douglas and Wormald 2006). However, with this injection technique the needle tip is located close to the maxillary artery, increasing the risk of intravascular injection. Stankiewicz (1988) recommends bending the needle at the hub to an angle of 45° and injecting it to a depth of 25–28 mm. Das et al. (2006) recommended bending the needle to a 60° angle and inserting it 25 mm into the GPC. Douglas and Wormald modified the injection technique by pre-bending the needle not only to a specified angle (45°) but also at a specific length (25 mm) and then inserting the needle to the bend. Infiltrating the GPC to a depth of about 25 mm is most probably too shallow for adequate anaesthesia, but is sufficiently deep to obtain adequate haemostasis for endoscopic sinus surgery (Douglas and Wormald 2006).

One has to acknowledge that both an adequate knowledge of GPC anatomy and the correct use of the chosen injection technique are crucial if complications are to be avoided. Although GPC injection is regarded as a rather safe procedure (Das et al. 2006), some authors have reported complication rates as high as 36 % (Wong and Sved 1991) for diplopia (secondary from diffusion of anaesthetic solution through the inferior orbital fissure) and 12 % for strabismus, as well as a 10 % rate of ptosis (secondary to anaesthesia of the oculomotor nerve). However, all of these complications were reported to be transient, and the clinician has therefore to weigh the risk of potential complications against the much greater complication risk of performing endoscopic sinus surgery with poor visualization because of bleeding (Das et al. 2006).

This study has also confirmed that the GPC, just like almost every other part of the human skull, is subject to sexual dimorphism. Sexual dimorphism of the greater palatine canal has been previously reported by Sheikhi et al. (2013), and we agree that the craniofacial complex is highly variable in both size and shape by sex and that the zygomatic curve and skull size are generally larger in males than in females (Bigoni et al. 2010). In a previous study (Tomaszewska et al. 2014b) we found that the length of the GPC, among other variables, can be successfully used to distinguish between sexes, with an overall accuracy of >78 %. In the present study we found a number of rather discrete side-related differences in both males (GPF lateral-medial dimension) and females (GPC length). These differences were expected as asymmetry is common in craniofacial bones. Inconsistencies in the growth of the right and left GPC could be due to genetic and/or environmental factors. Asymmetric expression of craniofacial features is also related to the functional activity of the musculoskeletal system—in this case, specifically the masticatory apparatus (Rossi et al. 2003). Side-related cranial discrepancies are also subject to demographic changes resulting from human migration, which has increased in the last two centuries in particular (most seen in North America and Central Europe) (Jonke et al. 2007). Such demographic shifts could also account for the changes seen in this study as the population examined is that of typical Central European Caucasians. Another potential explanation for the discovered side-related differences includes the manner in which the palate develops, as this process is dependent on the function of several ossification centres (Slavkin et al. 1966).

Previous to the present study, only two studies (Howard-Swirzinski et al. 2010; Sheikhi et al. 2013) analysed in detail GPC pathway types in both the sagittal and coronal planes. We agree with the authors of these two studies regarding the types of GPC pathways, but we found major differences in terms of incidence. In our study, the most common GPC path in the sagittal plane was the same as that reported by Sheikhi et al. (2013), but it differed from that reported by Howard-Swirzinski et al. (2010), who found that the most common GPC pathway type observed in our study was overall second, but at an incidence of only 6.5 %. As the present study is the largest performed to date, and the obtained results dispute those from the second largest study (*n* = 500 subjects; Howard-Swirzinski et al. 2010), we suggest that there is a definite need to perform further large retrospective CT-based studies that would analyse GPC pathway types and their incidence in different populations. In the coronal plane the results of all three studies can be considered similar. In terms of the angles by which the GPC deviates from a vertical line, in both the sagittal and coronal planes, and the length of specific GPF parts (Table 4), our results are similar to the ones obtained by Howard-Swirzinski et al. (2010). This similarity prevents us from justifying the differences in GPC pathway type observed in the sagittal plane using the palate ossification centre theory (Slavkin et al. 1966) and most probably points to the fact that these differences can be attributed to relatively small sample sizes of the analysed groups (when related to the general population) and selectivity bias.

The strong points of the present study include a large sample size (largest to date) and the inclusion of a systematic review (according to PRISMA guidelines) in terms of GPC length, GPF dimensions and opening direction. However, the systematic review is also the source of the largest limitation of the study—namely that the number of studies assessing the length of the GPC is very limited. We also have to mention that the retrospective nature of this study prohibited us from gathering additional morphometric data on the subjects enrolled in the study (e.g. stature).

In conclusion, a thorough understanding of GPC length and pathway types is needed to properly administer anaesthesia prior to maxillofacial procedures. Through a systematic review of literature and an extensive analysis of CT scans, we report data on GPC length and pathway types in a large Eastern European sample with reference to studies on different populations. The information presented here provides clinicians with the anatomical knowledge necessary to minimize the risk of complications when performing procedures involving infiltration of the GPC.

## References

[CR1] Ajmani ML (1994). Anatomical variation in position of the greater palatine foramen in the adult human skull. J Anat.

[CR2] Bigoni L, Velemínská J, Brůžek J (2010). Three-dimensional geometric morphometric analysis of cranio-facial sexual dimorphism in a Central European sample of known sex. J Comp Hum Biol.

[CR3] Chrcanovic BR, Custódio AL (2010). Anatomical variation in the position of the greater palatine foramen. J Oral Sci.

[CR4] Das S, Kim D, Cannon TY, Ebert CS, Senior BA (2006). High-resolution computed tomography analysis of the greater palatine canal. Am J Rhinol.

[CR5] Dave MR, Yagain VK, Anadkat S (2013). A study of the anatomical variations in the position of the greater palatine foramen in adult human skulls and its clinical significance. Int J Morphol.

[CR6] Douglas R, Wormald PJ (2006). Pterygopalatine fossa infiltration through the greater palatine foramen: where to bend the needle. Laryngoscope.

[CR7] Erdogan N, Unur E, Baykara M (2003). CT anatomy of pterygopalatine fossa and its communications: a pictorial review. Comput Med Imaging Graph.

[CR8] Hassanali J, Mwaniki D (1984). Palatal analysis and osteology of the hard palate of the Kenyan African skulls. Anat Rec.

[CR9] Howard-Swirzinski K, Edwards PC, Saini TS, Norton NS (2010) Length and geometric patterns of the greater palatine canal observed in cone beam computed tomography. Int J Dent pii:292753. doi:10.1155/2010/292753 10.1155/2010/292753 PMC2943084 20871845

[CR10] Hwang SH, Seo JH, Joo YH, Kim BG, Cho JH, Kang JM (2011). An anatomic study using three-dimensional reconstruction for pterygopalatine fossa infiltration via the greater palatine canal. Clin Anat.

[CR11] Ikuta CR, Cardoso CL, Ferreira-Júnior O, Lauris JR, Souza PH, Rubira-Bullen IR (2013). Position of the greater palatine foramen: an anatomical study through cone beam computed tomography images. Surg Radiol Anat.

[CR12] Jaffar AA, Hamadah HJ (2003). An analysis of the position of the greater palatine foramen. J Basic Med Sci.

[CR13] Jonke E, Prossinger H, Bookstein FL, Schaefer K, Bernhard M, Freudenthaer JW (2007). Secular trends in the facial skull from the 19th century to the present analyzed with geometric morphometrics. Am J Orthod Dentofac Orthop.

[CR14] Klosek SK, Rungruang T (2009). Anatomical study of the greater palatine artery and related structures of the palatal vault: considerations for palate as the subepithelial connective tissue graft donor site. Surg Radiol Anat.

[CR15] Kumar A, Sharma A, Singh P (2011). Assessment of the relative location of greater palatine foramen in adult Indian skulls: consideration for maxillary nerve block. Eur J Anat.

[CR16] Langenegger JJ, Lownie JF, Cleaton-Jones PE (1983). The relationship of the greater palatine foramen to the molar teeth and pterygoid hamulus in human skulls. J Dent.

[CR17] Malamed SF, Trieger N (1983). Intraoral maxillary nerve block: an anatomical and clinical study. Anesth Prog.

[CR18] McKinney KA, Stadler ME, Wong YT (2010). Transpalatal greater palatine canal injection: radioanatomic analysis of where to bend the needle for pediatric sinus surgery. Am J Rhinol Allergy.

[CR19] Mercuri LG (1979). Intraoral second division nerve block. Oral Surg Oral Med Oral Pathol.

[CR20] Methathrathip D, Apinhasmit W, Chompoopong S, Lertsirithong A, Ariyawatkul T, Sangvichien S (2005). Anatomy of greater palatine foramen and canal and pterygopalatine fossa in Thais: considerations for maxillary nerve block. Surg Radiol Anat.

[CR21] Nimigean V, Nimigean VR, Buţincu L, Sălăvăstru DI, Podoleanu L (2013). Anatomical and clinical considerations regarding the greater palatine foramen. Rom J Morphol Embryol.

[CR22] Oluigbo CO, Makonnen G, Narouze S, Rezai AR (2011). Sphenopalatine ganglion interventions: technical aspects and application. Prog Neurol Surg.

[CR23] Osunwoke EA, Amah-Tariah FS, Bob-Manuel IF, Nwankoala QK (2011). A study of the palatine formane in dry human skulls in south–south Nigeria. Scientia Africana.

[CR24] Piagkou M, Xanthos T, Anagnostopoulou S (2012). Anatomical variation and morphology in the position of the palatine foramina in adult human skulls from Greece. J Craniomaxillofac Surg.

[CR25] Rossi M, Ribeiro E, Smith R (2003). Craniofacial asymmetry in development: an anatomical study. Angle Orthod.

[CR26] Saralaya V, Nayak SR (2007). The relative position of the greater palatine foramen in dry Indian skulls. Singap Med J.

[CR27] Sharma NA, Garud RS (2013). Greater palatine foramen–key to successful hemimaxillary anaesthesia: a morphometric study and report of a rare aberration. Singap Med J.

[CR28] Sheikhi M, Zamaninaser A, Jalalian F (2013). Length and anatomic routes of the greater palatine canal as observed by cone beam computed tomography. Dent Res J (Isfahan).

[CR29] Slavkin HC, Canter MR, Canter SR (1966). An anatomic study of the pterygomaxillary region in the craniums of infants and children. Oral Surg.

[CR30] Stankiewicz JA (1988). Greater palatine foramen injection made easy. Laryngoscope.

[CR31] Sved AM, Wong JD, Donkor P (1992). Complications associated with maxillary nerve block anaesthesia via the greater palatine canal. Aust Dent J.

[CR32] Tomaszewska IM, Tomaszewski KA, Kmiotek EK (2014). Anatomical landmarks for the localization of the greater palatine foramen—a study of 1200 head CTs, 150 dry skulls, systematic review of literature and meta-analysis. J Anat.

[CR33] Tomaszewska IM, Frączek P, Gomulska M (2014). Sex determination based on the analysis of a contemporary Polish population’s palatine bones: a computed tomography study of 1,200 patients. Folia Morphol (Warsz).

[CR34] Viegas AR, Hemphill FM (1961). ) Predicting depth of insertion of needle required to anesthetize the maxillary nerve by way of the pterygopalatine canal. J Oral Surg.

[CR35] Wang TM, Kuo KJ, Shih C, Ho LL, Liu JC (1988). Assessment of the relative locations of the greater palatine foramen in adult Chinese skulls. Acta Anat (Basel).

[CR36] Westmoreland EE, Blanton PL (1982). An analysis of the variations in position of the greater palatine foramen in the adult human skull. Anat Rec.

[CR37] Wong JD, Sved AM (1991). Maxillary nerve block anaesthesia via the greater palatine canal: a modified technique and case reports. Aust Dent J.

